# Cytokinin Response Factor 9 Represses Cytokinin Responses in Flower Development

**DOI:** 10.3390/ijms24054380

**Published:** 2023-02-22

**Authors:** Christine Swinka, Eva Hellmann, Paul Zwack, Ramya Banda, Aaron M. Rashotte, Alexander Heyl

**Affiliations:** 1 Institut für Angewandte Genetik, Freie Universität Berlin, Albrecht Thaer Weg 6, 14195 Berlin, Germany; 2 Department of Biological Sciences, Auburn University, 101 Rouse Life Sciences, Auburn, AL 36849, USA; 3 Department of Biology, Adelphi University, 1 South Ave, Garden City, NY 11530, USA

**Keywords:** cytokinin, Cytokinin Response Factor, transcription, repressor, flower development

## Abstract

A multi-step phosphorelay system is the main conduit of cytokinin signal transduction. However, several groups of additional factors that also play a role in this signaling pathway have been found—among them the Cytokinin Response Factors (CRFs). In a genetic screen, CRF9 was identified as a regulator of the transcriptional cytokinin response. It is mainly expressed in flowers. Mutational analysis indicates that CRF9 plays a role in the transition from vegetative to reproductive growth and silique development. The CRF9 protein is localized in the nucleus and functions as a transcriptional repressor of *Arabidopsis Response Regulator 6* (*ARR6***)**—a primary response gene for cytokinin signaling. The experimental data suggest that CRF9 functions as a repressor of cytokinin during reproductive development.

## 1. Introduction

The plant hormone cytokinin regulates numerous developmental processes throughout the plant, from early events in embryogenesis to reproductive development and leaf senescence [1,2,3,4,5]. The Two-Component Signaling pathway (TCS) is a modified version of the bacterial two-component system consisting of receptor histidine kinases, phosphotransfer proteins, and two classes of Response Regulators (RRs) [5,6,7,8,9]. Type-B RRs (RRBs) are transcription factors that positively regulate responses to cytokinin, while type-A RRs lack DNA binding activity and act as negative regulators [6,10]. Although these key components of the TCS pathway have been well characterized in *Arabidopsis* and other species, many of the downstream regulatory mechanisms of the cytokinin responses remain unclear [3,8,10]. A small subgroup of AP2/ERF family transcription factors, known as Cytokinin Response Factors or CRFs, has been shown to be involved in the regulation of cytokinin signaling and response [11,12,13,14,15].

CRFs have been shown to act in concert with RRBs in the transcriptional regulation of cytokinin-responsive genes and are involved in protein–protein interactions with components of the primary signal transduction pathway [11,16,17]. Functional characterizations of CRFs have implicated these transcription factors in the regulation of multiple processes related to shoot growth and development including leaf and cotyledon expansion, leaf vascular patterning, and senescence [11,17,18]. There have also been connections between CRFs and an array of abiotic stress responses in addition to development, which was reviewed in [12,13,14,19]. These are in line with similar findings that have generally connected cytokinin to different stress responses [20,21].

The *CRFs* were first identified in a screen for cytokinin-responsive genes in *Arabidopsis* as related *AP2/ERF* transcription factor genes [13]. These were later, named and specifically described as *CRFs*, including three *CRFs* (*CRFs 2*, *5*, and *6*) that are members of a group of genes that show strong transcriptional induction by cytokinin treatment [11,12,22,23]. Additional members of the *CRF* group (Clade VI, VI-L—ERFs) were identified later, yielding a total of 12 *CRFs* in *Arabidopsis* (*CRF1-12*) [11,12,17,24,25].

Examinations of CRF protein and gene sequences across Angiosperm species have revealed that the *CRF* group can be generally spilt into five distinct phylogenetic clades (I to V) [12,17]. In nearly every diploid Angiosperm species examined, there are either 1 or 2 genes found per *CRF*-Clade, with expected increases in polyploid plants (i.e., 2–4 in a tetraploid). However, Clade V *CRF* groups routinely have 3–4 genes in diploid species examined. Primary examples of species where there have been full examinations of *CRFs* include tomato (3 Clade V genes: *SlCRF9*, *10*, *11*) and *Arabidopsis* (Four Clade V genes: *AtCRF9*, *10*, *11*, *12*). Further evidence from the gathering of additional Angiosperm *CRF* sequence phylogenetic examinations, suggests that Clade V should likely be split into two distinct clades or at least subclade Clade Va and Vb, with *AtCRF9*, *SlCRF9*, and their orthologs in one group Va and the other Clade V members in a distinct group Vb, *AtCRF10*, *11*, *12*, and *SlCRF10*, *11* [18,26].

In a genetic screen to identify novel transcriptional regulators of the cytokinin response in *Arabidopsis*, CRF9 was found to regulate the transcription of the reporter gene *ARR6* [27]. This study aimed to functionally characterize CRF9. It was demonstrated that this gene acts to repress cytokinin responses. Additionally, we show that CRF9 is a direct regulator of the *ARR6* expression.

## 2. Results

### 2.1. CRFs Are Found in Early Divergent Land Plants and Streptophyte Algae

**Figure 1:**
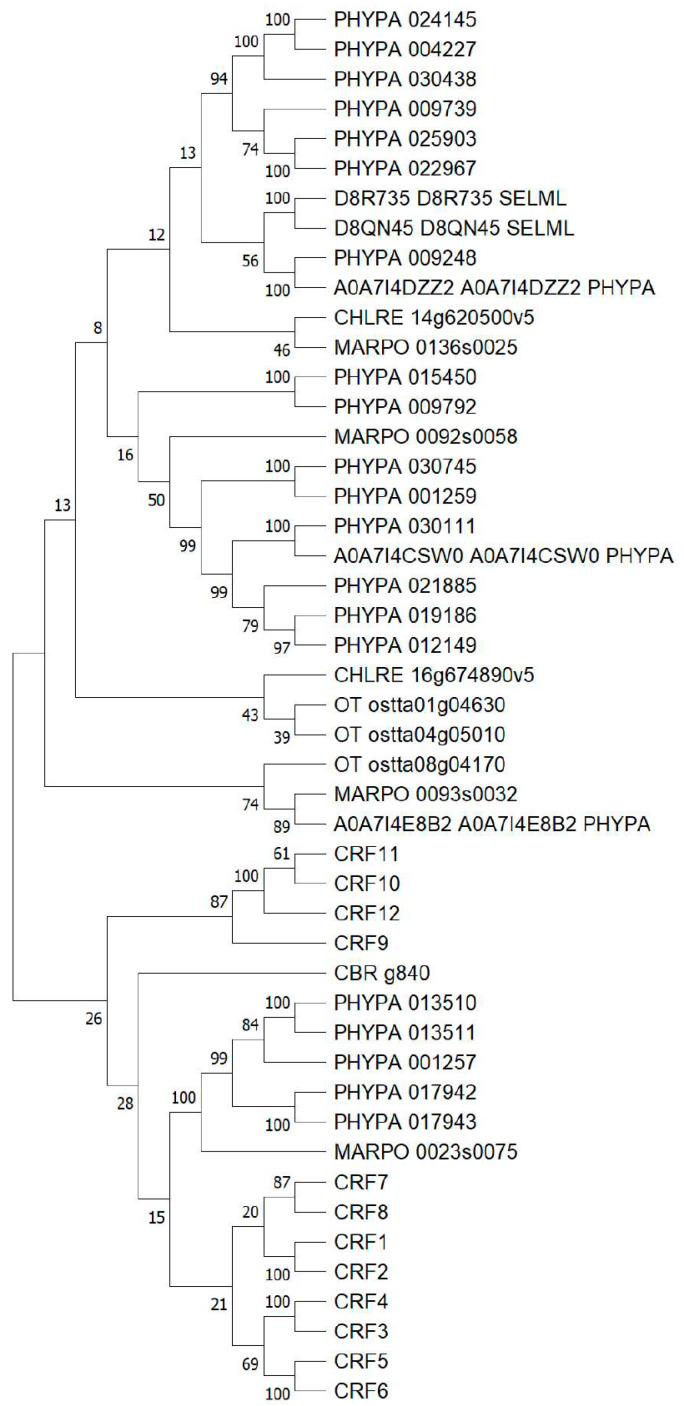
Phylogenetic analysis of CRFs in early diverging land plants and algae. CRF9 of *Arabidopsis* was used as a query in a HMMR search to find homologs in different plant species (*Ostreococcucs taurii* OT, *Chlamydomonas rheinhardtii* CHLRE, *Klebsormodium nitans*, *Chara braunii* CBR, *Mesotigma viride*, *Marchantia polymorpha* MARPO, *Physcomitrium patens* PHYPA, *Selaginella moelendorfii* SELML). After a quality check, the resulting positives were aligned using the MAFFT program, and a Maximum Likelihood tree was calculated based on the resulting alignment. The quality of the tree was evaluated using 200 bootstrap repetitions.

While the phylogeny of CRFs is well established in modern land plants [25], the origin of this protein family is not clear. To gain some insights, a phylogenetic analysis targeting early land plants and algae was conducted. The results show that all known CRFs from the *Arabidopsis* clade in one branch are clearly different from sequences of most proteins (Figure 1). Within this branch of the tree, there is also one sequence from *M. polymorpha* and four from *P. patens*, the two most early diverging land plants in this analysis. Interestingly, the only sequence of Charophyceae algae, *C. braunii*, is also found in this branch. All other sequences are distinct and probably represent members of the AP2/ERF family, which are not CRFs. No members of the CRF family were found in Chlorophyceae algae or the other Charophyceae algae included in this analysis.

### 2.2. CRF9 Is Mainly Expressed in the Flower

**Figure 2:**
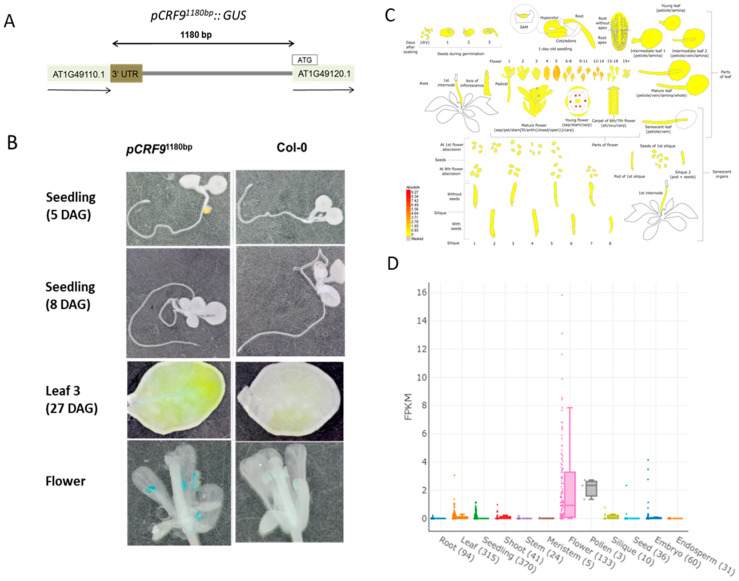
Expression analysis of *CRF9.* The expression of *CRF9* was analyzed. (**A**) Tissue level expression for *CRF9* was determined by fusing the promoter (1180 bp upstream of the ATG) to a GUS reporter construct and generating stable transgenic reporter lines. (**B**) Transgenic lines were examined by GUS staining across different stages of development, where the primary expression occurred in flower anthers. (**C**,**D**) Additional examination of *CRF9* expression from RNA-sequencing experiment—The Klepikova developmental data set from eBAR (**C**) and an accumulation of over 20,000+ RNA-seq experiments—the *Arabidopsis* RNA-seq Database (**D**) also reveal similar patterns of the highest expression occurring in flower anthers and pollen. DAG = Day after germination.

To learn about the biological function of *CRF9*, the expression of the gene was analyzed using a promoter–*GUS* construct (Figure 2A). This construct used the whole *CRF9* upstream region and the 3′UTR of the next gene upstream AT1G49110 (in a total length of 1180 bp). The results showed GUS expression in the flower, primarily the anthers with a small amount of expression in the leaf mid-vein at 27 DAG (Figure 2B). Publicity available RNA-seq data confirmed that the expression pattern of *CRF9* expression was primarily localized to RNA samples collected from flowers and pollen (Figure 2C,D).

In summary, the expression analysis using GUS and RNA seq data demonstrates *CRF9* expression mainly in the reproductive tissues.

### 2.3. The Putative Transcription Factor CRF9 Is Located in the Nucleus

**Figure 3:**
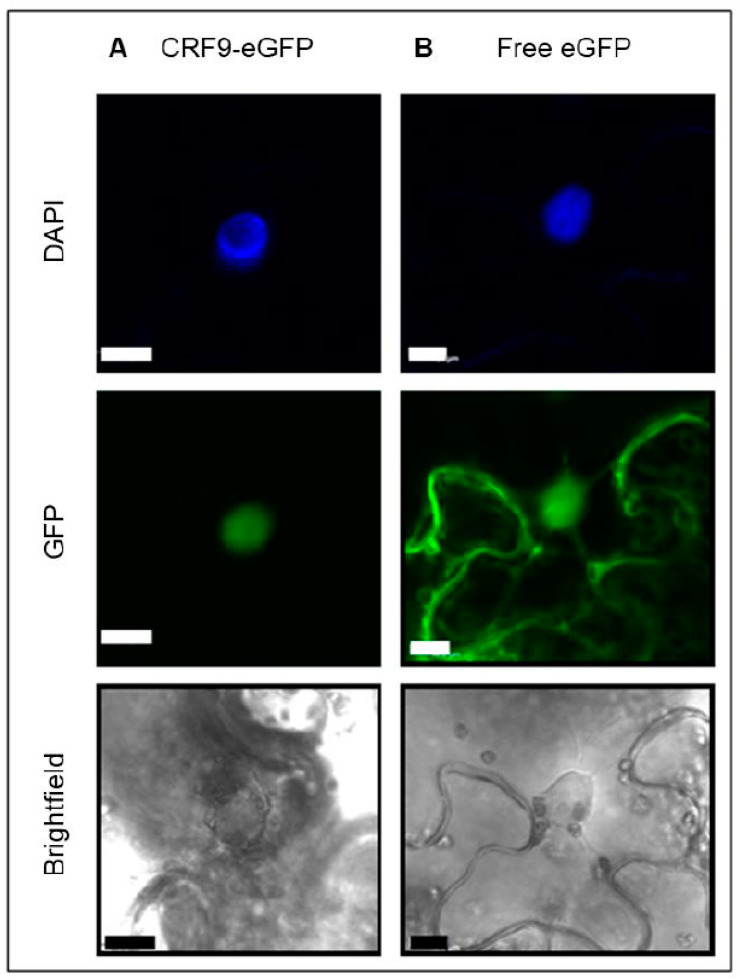
CRF9 is localized in the nucleus. The subcellular localization of CRF9 was determined by fusing the gene to an eGFP moiety and transiently transforming tobacco leaf disk with these constructs. After 48 h, the eGFP fluorescence was determined with a confocal microscope. The eGFP was excited at 488 nm and the emitted radiation was detected between 500–600 nm. The nucleus was stained using DAPI and the signal was detected between 420–490 nm by using a 405 diode in the UV spectrum. Shown is the detected signal of the c-terminal fusion (**A**) CRF9-eGFP and (**B**) free eGFP which served as a negative control. The bar (white or black) represents a size of 10 µm.

The bioinformatics analysis places CRF9 in the AP/ERF transcription factor group [17]. Thus, we wanted to test if the protein indeed localizes to the nucleus. CRF9 N- and C-terminal GFP fusion constructs, driven by the 35S promoter, were made and used to transiently transform tobacco leaf cells. After a 48 h incubation period, the samples were analyzed by confocal microscopy. For both CRF9 GFP fusion proteins, strong signals exclusively in the nucleus were detected (Figure 3). The nuclear localization was later confirmed by a DAPI staining of the nucleus.

### 2.4. CRF9 Plays a Role in Plant Development

**Figure 4:**
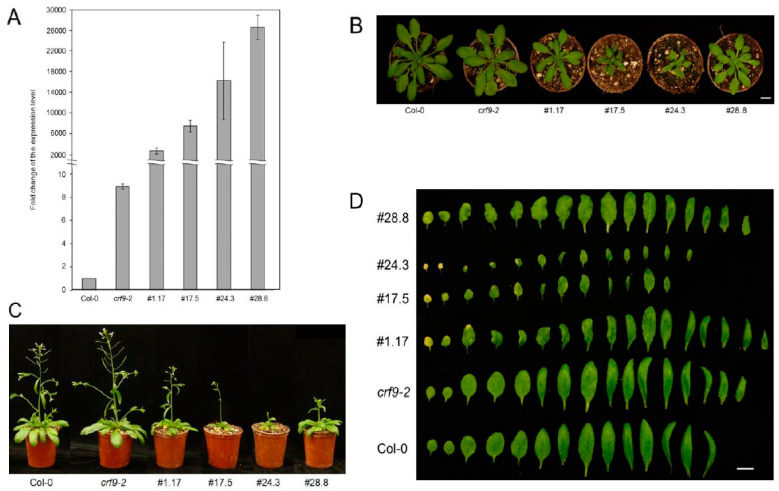
Analysis of the phenotype of *CRF9* mutants. Plants of the wild-type Col-0, *crf9-2*, and four independent *CRF9* overexpressing lines were grown at long-day conditions in the greenhouse. (**A**) The *CRF9* expression levels in 7-day-old seedlings were analyzed using qRT-PCR. (**B**,**C**) The shoot phenotype of the plants 28 days after germination. (**D**) Leaves of the different plant lines were used in this study at the time of floral transition.

**Figure 5:**
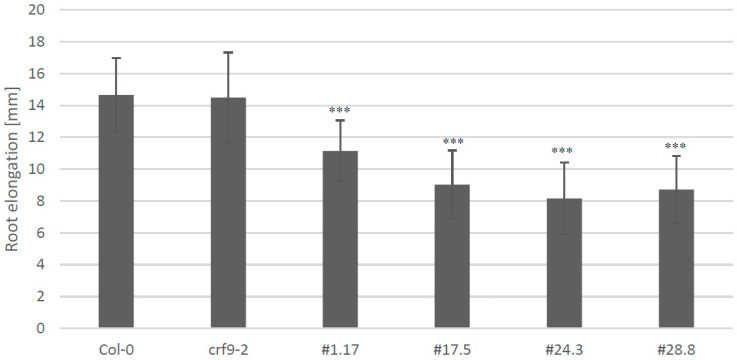
*CRF9* overexpression leads to a shorter primary root. Plants were grown on 0.5× MS media under long-day conditions in the growth chamber. The tip of the primary root was marked on the third and tenth day after germination and the root elongation was determined using the program ImageJ (www.imagej.nih.gov/ij/ accessed on 21 December 2022). Level of significance in a one-sided *t*-test: *** = *p* < 0.001.

To investigate the function of a gene in a given organism, the gene of interest can be either mutated or overexpressed. We chose both approaches and analyzed the phenotypes of a T-DNA insertion mutant, and four independent overexpressor lines (# 1.17; 17.5; 24.3; 28.8), using the 35S promoter to drive *CRF9* expression. The T-DNA insert of the knock-out mutant (*crf9-2*) was located at the C-terminal end of the gene downstream of the functional domains but within the Clade V C-terminal specific region. Surprisingly, qRT PCR revealed even higher levels of *CRF9* in this mutant line compared to the wild type. The same analysis demonstrated very high levels of *CRF9* transcripts in the overexpressor lines tested (Figure 4A). The *crf9-2* line showed a similar growth pattern as seen for wild-type plants. However, plants of the *CRF9* ox lines were to a varying degree smaller than the wild type (Figure 4B,C). Two of the overexpressor lines, 17.5 and 24.3, also had fewer and smaller leaves. The other two lines investigated showed a similar leaf size, but higher leaf number compared to the wild-type plants (Figure 4D). Next, the effect of *CRF9* on root growth was evaluated. Again, the *crf9-2* line did not significantly differ in root elongation from the wild type. In contrast, the roots of the *CRF9* overexpressor lines were significantly shorter than the wild-type, seemingly independent of the level of *CRF9* overexpression (Figure 5).

*CRF9* overexpression led to smaller plants compared to the wild-type, while the *crf9-2* line was very similar to the wild-type.

### 2.5. Reproductive Traits Are Affected by CRF9

**Figure 6:**
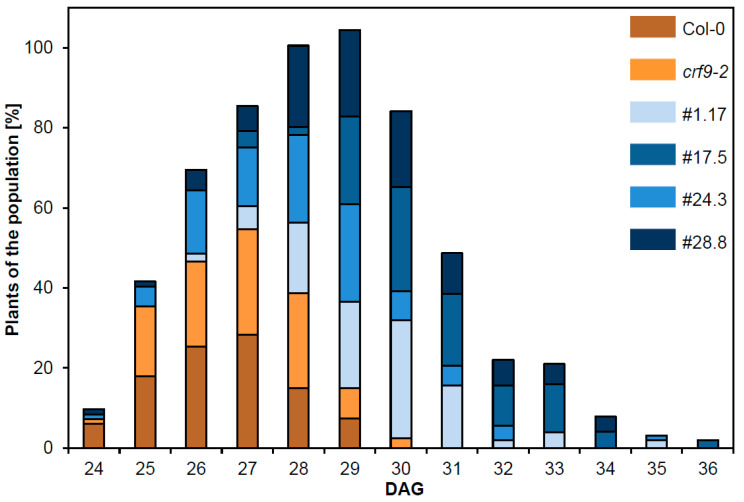
Overexpression of *CRF9* causes delayed transition from vegetative to reproductive growth. Plants of the different lines (*crf9-2*, the four *CRF9* overexpressing lines, and wild-type plants) were grown in the greenhouse under long-day conditions. The transition from vegetative to reproductive growth was determined as the time between germination and the time point when the stem was 1 cm in height (*n* ≥ 50).

After analyzing the effects of *CRF9* on vegetative development, we looked at the reproductive traits. The time to floral transition was determined in all plant lines and was defined as the age that the stem reached a height of longer than 1 cm. In both the wild-type and the *crf9-2* lines, the floral transition started 24 days after germination (DAG), and all plants had transitioned from vegetative to reproductive growth at 30 DAG. In contrast, the *CRF9*-overexpressing lines also started flowering or bolting at 24–26 DAG, but the transition took a longer time, as only at 36 DAG all plants had started their reproductive growth (Figure 6).

**Figure 7:**
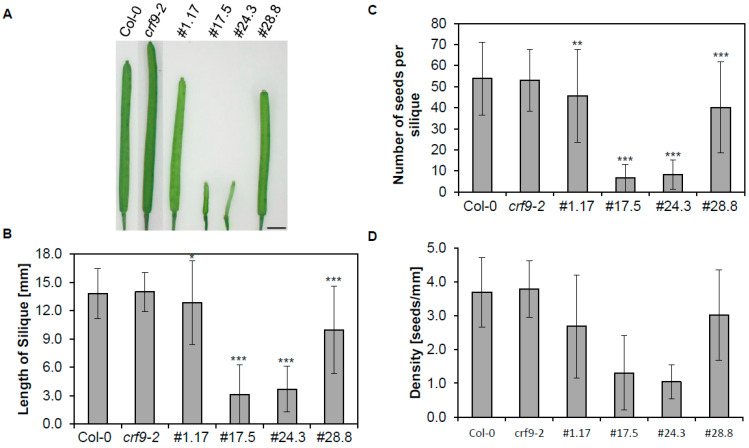
CRF9 seems to play a critical role in silique development. The different plant lines were grown under long-day conditions in the greenhouse. (**A**) Siliques at the end of their respective growth phase. (**B**) length of siliques (*n* ≥ 120) (**C**) the average number of seeds per silique (*n* ≥ 17) (**D**) seed density (number of seeds per mm of silique length (*n* ≥ 17). Level of significance in a one-sided *t*-test: * = *p* < 0.05, ** = *p*< 0.01, *** = *p* < 0.001. The rule bar in (**A**) represents 2 mm.

After fertilization, the resulting siliques of the *CRF9*-overexpressing lines were significantly shorter than those of wild-type and the *crf9-2* plants—with the strongest phenotype overserved again in lines 17.5 and 24.3 (Figure 7A,B). These reduced silique lengths resulted in lower seed density (seeds/mm of silique) as well as fewer seeds in total. The greatest reduction was detected in lines 17.5 and 24.3 with a gradual decrease in seed number in the other two overexpressing lines and no detectable difference between *crf9-2* and the wild-type plants (Figure 7C,D). However, no difference was detected concerning the size of the seeds themselves.

In summary, *CRF9*-overexpressing lines showed a later transition from vegetative to reproductive growth and their siliques were smaller and had a lower seed density.

### 2.6. CRF9 Affects the Cytokinin Response in Roots

**Figure 8:**
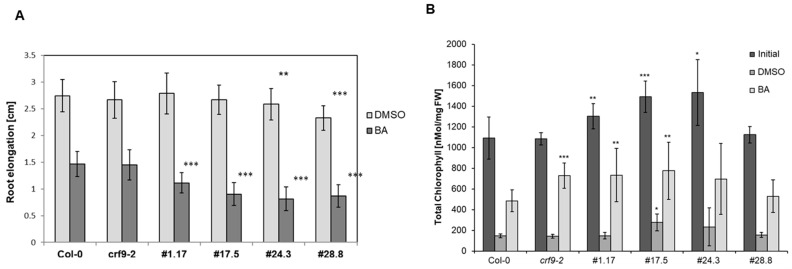
*CRF9* overexpression leads to attenuation of cytokinin effect in roots. The effect of *CRF9* overexpression was tested in two different long-term cytokinin response assays. (**A**) Plants of the different lines investigated were grown on 0.5× MS-media containing 100 nM benzyl adenine (BA) under long-day conditions in a growth chamber. DMSO was used as a solvent control. The tip of the primary root was marked on the third and tenth day after germination and the root elongation was determined using ImageJ (www.imagej.nih.gov/ij/, accessed on 21 December 2022). (**B**) The sixth leaves of the respective plants was taken and incubated for six days in the dark with 1 µM BA or DMSO as solvent control. After the sixth day, the chlorophyll of the dark-incubated leaves and those of untreated leaves was extracted and measured (*n* = 12 Level of significance in a one-sided *t*-test: * = *p* < 0.05, ** = *p* < 0.01, *** = *p* < 0.001.

Only some CRFs have been shown to be transcriptionally regulated by cytokinin and affect cytokinin response in plants [12]. Thus, the effect of CRF9 on the response to cytokinin was examined using standard bioassays. First, the effect of cytokinin on root elongation was tested. Cytokinin treatment did not lead to a detectable difference between the wild-type and *crf9-2* plants. In contrast, all the *CRF9*-overexpressing lines showed a significant reduction in root elongation after cytokinin treatment (Figure 8A). A similar picture emerged in a second, well-established cytokinin bioassay, the chlorophyll retention assay. The overexpressing lines showed a significant difference in chlorophyll content compared to the wild type (Figure 8B).

In summary, the *CRF9* overexpression led to detectable differences in long-term cytokinin response assays in both leaves and roots.

### 2.7. CRF9 Is a Transcriptional Repressor of ARR6

After detecting the effect of CRF9 overexpression on the cytokinin response on the whole plant level, the effects of the hormone on the molecular level were also investigated. Since *CRF9* is a member of the CRF transcription factor family, we looked at its effect on the expression of the well-characterized cytokinin response gene *ARR6*, a member of the RRA family using two different assays.

**Figure 9:**
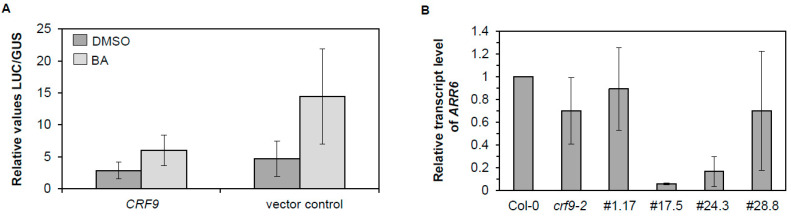
CRF9 acts as a transcriptional repressor of the cytokinin primary response gene *ARR6*. The molecular role of CRF9 was investigated using two assays. (**A**) In the protoplast transactivation assay, *35S::CRF9* represses the activation of the *ARR6::GUS* reporter gene. The activation of the *ARR6::GUS* reporter gene was measured without and 16 h after the addition of 500 nM *trans*-Zeatin (tZ). The *ARR6::GUS* reporter construct and a *35S::GUS* construct without any effector plasmid were used as controls. Protoplasts were cotransfected with the *ARR6::GUS* reporter and an effector plasmid expressing *CRF9*. Variations in transformation efficiencies were normalized using a *35S::NAN* reporter construct. The mean values and SD of four independent transfection assays were calculated and shown as relative GUS/NAN activity units. (**B**) The expression level of *ARR6* was determined in the different plant lines. Leaf 6, 7 or 10 of plants with 1 cm high stems were taken from the overexpressing lines and the wild type was grown for 30–36 days under long-day conditions in a growth chamber. The amount of *ARR6* transcript was determined using qRT-PCR.

In a protoplast transactivation assay (PTA) the effect of the expression of *CRF9* on the *ARR6* promoter was tested via a coupled *Luc* reporter gene [27]. The co-expression of *CRF9* led to a decrease in the *ARR6* promoter activity compared to the vector control, which would be consistent with *CRF9* functioning as a negative regulator of the molecular cytokinin response (Figure 9A). A similar picture emerged when the amount of *ARR6* transcripts were examined in the different plant lines. The *crf9-2* mutant lines and lines 1.17 and 28.8 displayed only weak or no reduction of the transcript of the primary response gene. In contrast, the strong *CRF9* overexpressor lines 17.5 and 24.3 displayed a dramatic reduction of *ARR6* mRNA (Figure 9B).

Thus, on the molecular level, *CRF9* acts as a negative regulator of at least one of the primary cytokinin response genes.

## 3. Discussion

It has long been known that the cytokinin response is mediated to a large extent by changes in transcriptional patterns [6,11,22,23,28,29]. RRBs and CRFs are the main regulators facilitating these changes in the transcriptome [12,16]. Since their original identification in *Arabidopsis* more than a decade ago, there have been several studies of some CRF genes as to their roles in development as well as cytokinin response, and environmental response in several different species [12,30,31]. Despite these efforts, there is still much that is not known about this group, and some phylogenetic clades of CRFs are basically unexamined. One such *CRF*, *CRF9* or At1g49120 (a Clade V CRF), which had not been studied in detail before, was identified in a genetic screen for transcriptional regulators [27]. This study aimed to functionally characterize this transcription factor.

### 3.1. CRF9 Plays a Role in Reproductive Growth

One of the best ways to study the function of any gene is to look for mutations. These can either be loss-of-function or gain-of-function mutations. In this study, both types of mutations have been utilized. In the different experiments shown here, the *crf9-2* mutant phenotype was not different from that of the wild type. This might be due to either redundancy among the *CRFs* or due to the allele that we used not leading to a loss of *CRF9* function. Only one prior report examined any *CRF9* mutant phenotype [17]. While that study did find some minor changes in leaf vascular patterning, no other phenotypes were reported, and a different T-DNA insertion mutant (*crf9-1*) was utilized. Loss-of-function mutations are often seen as more effective in characterizing the function of a gene. However, most modern land plants have undergone several rounds of whole genome duplications and consequentially show a high-level gene redundancy [32]. This high level of redundancy is also true for cytokinin-regulated transcription factors [33]. This could be a reason for the similarity of the phenotypes of *crf9-2* and wild-type plants. Another reason might be due to the position of the T-DNA insertion, downstream of the functional domains, or due to the level of *CRF9* transcript not being reduced but rather increased by the T-DNA insert (Figure 4A). In contrast, the overexpressor lines investigated displayed a distinctive phenotype (Figure 4). They had smaller or more leaves than the wild type, which would be consistent with the role of transcriptional repressor as a similar phenotype has been reported for *ARR1-SDRX*—a dominant repressor of the RRBs [34,35]. In addition, the later transition from vegetative to reproductive growth, which was detected in the overexpressor lines, has been reported before for *ARR1-SRDX* plants [34].

While those results are informative about the general function, the expression analysis showed the strongest expression for *CRF9* in the inflorescences and, thus, a biological function for the proteins is most likely to be found there. Interestingly, *CRF9* overexpressor lines showed significantly smaller siliques and a lower density (seeds/length of the silique) than the wild type (Figure 7), indicating a function for *CRF9* as a negative regulator for inflorescences development. This would be comparable to what has been reported for *ARR1-SRDX* plants [34]. The opposite phenotype was reported when the cytokinin content was increased, e.g., by mutating cytokinin metabolizing enzymes (*CKX*), as the siliques of those plants become longer [35,36].

### 3.2. CRF9 Is a Transcriptional Regulator of the Cytokinin Response

So how does CRF9 function on the molecular level? It was identified in a genetic screen to be a repressor of the primary cytokinin response gene *ARR6* [27]. The detected nuclear localization would be consistent with a function as a transcriptional regulator (Figure 3). The protoplast transactivation assay (PTA) has long been used to investigate the function of putative transcription factors involved in cytokinin signaling [6,27,34]. Our PTA experiment validates that CRF9 regulates *ARR6* as a repressor (Figure 9A). Furthermore, the expression analysis of *ARR6* in the different *CRF9* overexpressor lines confirmed a role for CRF9 as a negative regulator of this cytokinin primary response gene. Interestingly, other CRFs (CRF2, 3, and 6) have been described as directly targeting and inducing the auxin transport protein PIN1, which also has been linked to female reproductive organ developmental processes [37,38].

### 3.3. CRF9 Belongs to a Branch of CRFs Specific for Modern Land Plants

The phylogenetic analysis conducted in this study focused on the early diverging land species and green algae (Figure 1). The CRFs of *Arabidopsis* can be roughly separated into two groups (CRF1-CRF8 and CRF9-CRF12) as noted before [25]. The second group (B-clade) was further defined as CRF Clade V in a more detailed examination of CRFs [17]. Clade V seems to be specific for modern land plants as the CRFs from early diverging land plants and algae only clade with CRF1-CRF8. Given that CRF9 seems to play a role in flower development and flowers are an evolutionary novelty of modern land plants, it is tempting to speculate that the CRF9 branch is a result of an early whole genome duplication event and was neo-functionalized to function in the regulation of reproductive growth. However, more research is needed to support this hypothesis.

## 4. Materials and Methods

### 4.1. Phylogenetic Analysis

The protein CRF9 was used as a query in HMMR in *Plant Ensembl* (plants.ensembl.org) [39] to identify homologs in different plant species (*Ostreococcucs taurii*, *Chlamydomonas rheinhardtii*, *Klebsormodium nitans*, *Chara braunii*, *Mesotigma viride*, *Marchantia polymorpha*, *Physcomitrium patens*, *Selaginella moelendorfii*). All sequences that had an E-value of >1^−10^ and did not start with a Met were eliminated. All remaining sequences were checked for the presence of an AP2/ERF domain using Interpro (https://www.ebi.ac.uk/interpro, accessed on 21 December 2022) [40]. An alignment of the sequences was performed using MAFFT (www.ebi.ac.uk/Tools/msa/mafft, accessed on 21 December 2022) in the default setting [41]. The resulting alignment was imported into MEGA XI (www.megasoftware.net, accessed on 21 December 2022) [42]. A Maximum Likelihood tree was calculated using the JTT + G + I + F substitution setting. The resulting tree was evaluated using 200 bootstrap repetitions.

### 4.2. Plant Materials and Growth Conditions

Plants of the Columbia (Col-0) ecotype of *Arabidopsis thaliana* and *Nicotiana benthamiana* were used. For the analysis of the *CRF9* (At1g49120) function the mutant line GABI-Kat GK-351-H05, *crf9-2* was used [43]. Plants for the experiments were grown under long-day conditions in the greenhouse or a climate chamber at 21 °C.

### 4.3. Transgenic Plants

To generate transgenic lines for different purposes, the desired sequences were shuttled into vectors indicated using the GATEWAY system (Invitrogen^TM^). Wild-type plants from Col-0 were transfected by floral dip [44]. All used plant lines were homozygous for the T-DNA insertion. For overexpression studies, *CRF9* was cloned into the vector *pB2GW7* and for reporter studies, the promoter (1180 bp) of *CRF9* was shuttled into the *pBGWFS7* vector.

### 4.4. Subcellular Localization in Nicotiana Benthamiana

For subcellular localization studies, *CRF9* was shuttled into the vector *pB7WGF2* and *pB7FWG2* to generate N- and C-terminal fusion proteins with GFP using the GATEWAY^TM^ system. Plants from *N. benthamiana* were infiltrated with an agrobacteria solution containing the constructs for transient protein expression. Plants were incubated in the greenhouse for 2–3 days. For DAPI staining [45] leaf disks were punched out, put in a DAPI staining solution and vacuumed, followed by a 15 min incubation at 37 °C. The fluorescent signal from both DAPI and GFP was detected using a confocal microscope.

### 4.5. Root Elongation Assay

*Arabidopsis* seeds were surface sterilized with sodium hypochlorite solution and vertically grown on 0.5× MS medium on square plates. For cytokinin treatment, a medium containing 100 nM BA was used. Plates with an equal DMSO supplement served as a control. The position of the root tip was marked on day three and day ten after germination. The root elongation was determined using the program Image J (https://imagej.nih.gov/ij/index.html, accessed on 21 December 2022).

### 4.6. Histochemical Analysis

The respective plant tissues were stained with GUS staining solution as published before [46] and incubated overnight at 37 °C. After discarding the staining solution, the tissues were destained with 70% ethanol. The ethanol step was repeated several times until the tissue was cleared.

### 4.7. Dark-Induced Senescence Analysis

Leaf three and leaf four of 21 days old plants were analyzed for their chlorophyll content as described previously [47]. For lines #17.5 and #24.3, leaves five to eight were analyzed because leaves three and four already showed the early beginning of senescence. The initial chlorophyll content and the content after dark incubation were measured. The leaves were incubated in the dark for six days in an MES buffer containing 1 µM BA or DMSO as control. Three repetitions were performed.

### 4.8. Protoplast Transactivation Assay

*Arabidopsis* plants from the ecotype Col-0 were grown in a growth chamber under short-day conditions (8 h light and 16 h dark) and low light at 21 °C. Protoplasts were isolated from leaves five to ten from six-week-old plants by using the Tape-*Arabidopsis* Sandwich method [48]. The transfection of the protoplasts was performed following the description from Yoo [49] with some modifications. Protoplasts were transfected with three plasmids expressing the specific reporter *pARR6 2,4 kb::LUC* [6], an effector and the transfection control reporter pUbi::GUS [50]. The plasmids were used at the ratio 5:4:1. The double amount of each component of the transfection step was used to obtain higher values at the end. For cytokinin treatment, the transfected protoplasts were incubated overnight in the dark at 23 °C for around 16 h in 1 mL WI solution containing 500 nM trans-Zeatin. After that, LUC activity was analyzed following the instructions of the manufacturer (Promega Kit E1500) with the following modifications. For measuring the luminescence 50 µL of the protoplast solution was mixed with 50 µL Luciferase-Agent in a black 96-well plate. To analyze the transfection efficiency of each sample, 10 µL protoplast solution was assayed with 100 µL MUG solution (1 mM MUG, 10 mM Tris ph 8.0, 2 mM MgCl_2_). A kinetic read over 15 min was performed and the slope of the values was calculated. The activities were calculated as relative LUC/GUS values. The protoplast transactivation assay was performed as described before [27].

### 4.9. Floral Transition Analysis

For the analysis of the time point of transition from vegetative growth to reproduction growth, about 50 plants for each line were analyzed. The days as well as the number of leaves produced were counted once the stem reached 1 cm high.

## 5. Conclusions

In summary, the results of our experiments indicate that *CRF9* is a repressor of the cytokinin response in inflorescences. This would make this transcriptional regulator one of the few examples of negative regulators of the transcriptional response to cytokinin known today.

However, further experiments are needed to determine to which extent CRF9 controls other primary cytokinin response genes using gene editing to create a CRF9 functional knock-out and RNA-sequencing to see the effect on the floral transcriptome.
